# Application of DNA-Binding Protein Prediction Based on Graph Convolutional Network and Contact Map

**DOI:** 10.1155/2022/9044793

**Published:** 2022-01-17

**Authors:** Weizhong Lu, Nan Zhou, Yijie Ding, Hongjie Wu, Yu Zhang, Qiming Fu, Haiou Li

**Affiliations:** ^1^School of Electronic and Information Engineering, Suzhou University of Science and Technology, Suzhou, China; ^2^Provincial Key Laboratory for Computer Information Processing Technology, Soochow University, Suzhou, China; ^3^Suzhou Industrial Park Institute of Services Outsourcing, Suzhou, China

## Abstract

DNA contains the genetic information for the synthesis of proteins and RNA, and it is an indispensable substance in living organisms. DNA-binding proteins are an enzyme, which can bind with DNA to produce complex proteins, and play an important role in the functions of a variety of biological molecules. With the continuous development of deep learning, the introduction of deep learning into DNA-binding proteins for prediction is conducive to improving the speed and accuracy of DNA-binding protein recognition. In this study, the features and structures of proteins were used to obtain their representations through graph convolutional networks. A protein prediction model based on graph convolutional network and contact map was proposed. The method had some advantages by testing various indexes of PDB14189 and PDB2272 on the benchmark dataset.

## 1. Introduction

The sequence of a protein determines its structure and different structures determine different functions. There is about 18% of the weight of protein in the human body. As the carrier of life, it plays a very important role in human production and life. As a major component of life, proteins are involved in almost all activities of cells, including DNA replication and transcription, chromatin formation, cell growth, and a series of activities, all of which cannot be separated by specific proteins [1]. These proteins that bind to and interact with DNA are called DNA-binding proteins. It has a strong affinity with single-stranded DNA, but a small affinity with double-stranded DNA. Therefore, DNA-binding proteins are also called helical instability proteins, single-stranded DNA-binding proteins [2].

With the development of gene sequencing, various sequencing studies have left many DNA and proteins, including DNA-binding proteins. Using machine learning and deep learning methods to predict DNA-binding proteins has reached a good level, but there is still room for improvement.

At present, many methods based on machine learning have emerged to distinguish DNA-binding proteins, which are divided into structure and sequence methods. Yubo et al. [3] proposed a DBD-Hunter method that combines structural comparison with an assessment of statistical potential to measure the interaction between DNA bases and protein residues. Zhou et al. [4] used random forest for classification by adopting amino acid preservation pattern, potential electrostatic, and other features. However, these methods are too dependent on the protein structure, so the practical operation is difficult. Therefore, sequence-based studies were carried out. Liu et al. [5] proposed a new method for predicting DNA-binding proteins, IDNA-Pro, by integrating features into pseudoamino acids from protein sequences and classifying them through random forest. Zhao et al. [6] classified DNA-binding proteins based on the physicochemical properties of amino acids by using random forest to recognize the sequence features generated by PseAcc. Although the method based on machine learning can identify DNA-binding proteins well, it needs a lot of human intervention in the process of feature selection and could not properly grasp the relationship between data and features. To overcome this difficulty, deep learning techniques were introduced into protein prediction. Loo et al. [7] proposed a new prediction method MsDBP, which input the fused multiscale features into a deep neural network for learning and classification. The classification was tested with 67% accuracy on a separate dataset PDB2272. Compared with machine learning method, it can save the necessary manual intervention, but the prediction result needs to be improved.

**Figure 1:**
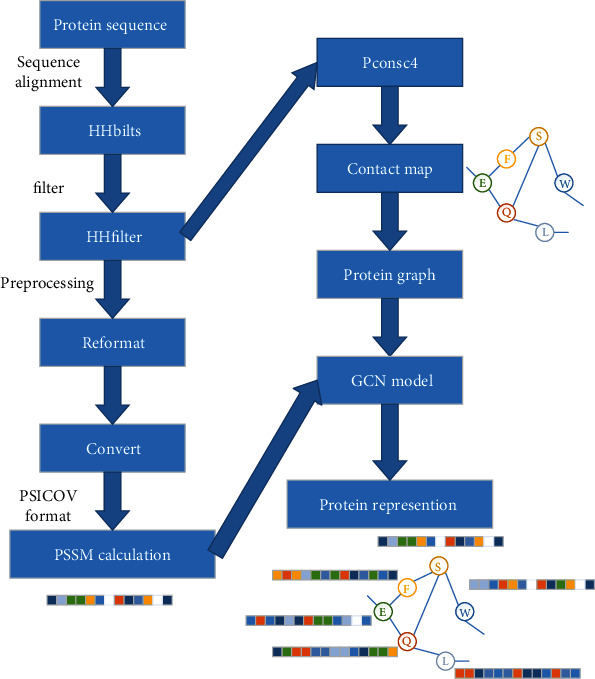
The processing of proteins, including the preprocessing of sequence, the generation of graph structures, and feature extraction, Pconsc4 was used to extract protein structural information. Finally, protein graph was generated higher-level feature graph through GCN.

Although there are many methods used to predict DNA-binding proteins at present, the results still have room for improvement. The main problem is how to obtain the high-precision protein structure from the protein sequence, because the accuracy of protein structure and feature has a great impact on the prediction results. In addition, the graph convolution network (GCN) has been widely used in the research of bioinformatics. Graph composed of nodes and edges serves as the input of the network without any requirements on size and format [8]. In order to improve the accuracy of structure and prediction, combining with the current developing trend of the technology of deep learning, a DNA-binding protein prediction model based on GCN and contact map was proposed. The protein graph depends on the sequence of the results of the comparison, so first introducing the preprocess of the dataset, including sequence comparison and filtering; the part of the output is used to calculate the features, and the other part as the input of Pconsc4 model [9], which is used to predict protein contact map, so the inputs of the model are feature matrix and adjacency matrix. We use them for training and prediction. The experimental results show that the prediction performance of DNA-binding proteins can be obtained by the method described. The research content of this paper is shown in Figure 1.

## 2. Materials and Methods

The prediction of DNA-binding proteins is divided into three parts: data preprocessing, training model, and testing. GCN differs from neural networks in that it introduces a graph structure to represent proteins, which can better represent the structure of proteins. The main purpose of protein sequence preprocessing is to obtain the features and structures of proteins. For the protein processing, the contact map is obtained by predicting the sequence through Pconsc4, and its output exactly corresponds to the adjacency matrix of GCN [10].

### 2.1. The Dataset

The DNA-binding protein dataset selected is the internationally common dataset. PDB14189 and PDB2272 were established by Gomes et al. [11]. Among them, the PDB14189 dataset was divided into 7129 DNA-binding protein sequences and 7060 DNA-unbinding protein sequences, and the PDB2272 dataset was divided into 1153 DNA-binding proteins and 1119 nonbinding proteins. PDB14189 was taken as the training set and PDB2272 as the test set. The dataset is detailed in Table 1 below. Among them, positive represents DNA-binding proteins, while negative represents non-DNA-binding proteins.

### 2.2. Protein Representation

**Figure 2:**
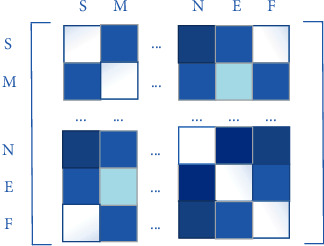
The contact map of protein.

The representation of proteins is generally divided into spatial structure and feature. The long-chain stable structure of protein also contains hydrogen bonds, hydrophobic bonds, salt bonds, and so on [12]. Each protein contains lots of atoms, if each atom is viewed as a node, then the protein graph will be very large, which will increase the pressure of training and is not easy to achieve. However, there are about hundreds of residues in a protein, and there is no other spatial information between residues, so it is more suitable to be used as nodes to represent structural features. The spatial structure of a protein can be represented by a contact map; it represents the two-dimensional structure of the protein; each element in the matrix represents the probability of contact at the corresponding position [13]; the value is between 0 and 1.  Figure 2 shows a protein contact map.

Predicting the structure of a protein from its sequence is the purpose of introducing contact map. Specifically, assuming that the length of protein sequence is *M*, the size of its contact map is *M*∗*M*. *M*(*i*, *j*) represents the probability of contact between the *i*th residue and the *j*th residue. If the value is less than the threshold value, it can be considered that they are in contact. Pconsc4 is a fast and efficient method to predict contact map. Since its output is a probability value between 0 and 1, the threshold value of 0.5 was set for the obtained contact maps, and the probability value greater than or equal to 0.5 was set as 1.The rest were set as 0, so that the structural information of the protein could be well extracted, corresponding to the adjacency matrix as the input GCN network [14].

The next step is the extraction of protein features. Since residues are used as nodes, the properties of residues are selected as features. Due to the differences in the R group, different features are displayed, including aromaticity, polarity, and explicit valence [15]. Position-specific scoring matrix (PSSM) is a commonly used representation of protein features, in which the results of each element depend on the results of sequence comparison, and these results represent the feature of proteins [16]. Other features were also used, such as the primary thermal coding of the remaining symbols, whether the residue was aromatic, whether the residue was acidic charged, and whether it was extremely neutral, etc. [17], as shown in Table 2. In summary, the total number of features is 54, so the protein's feature matrix dimension is (*M*, 54).

For PSSM, the basic position frequency matrix (PFM) [18] is calculated by the number of occurrence of residues at each position in the sequence of sequence alignment results. Equation (1) is as follows:

(1) $$\begin{array}{c} M_{k,j}^{\text{PFM}}=\overset{N}{\underset{i=1}{\sum }}I\left(A_{i,j}=k\right), \end{array}$$

where *A* represents a set of alignment sequences equal to the target protein length, *k* is the set of residues, *i* = (1, 2 ⋯ , *N*), *j* = (1, 2, ⋯*L*), and *i*(*x*) is the indicator function when the condition is met or not. Equation (2) is used to obtain the position probability matrix (PPM):

(2) $$\begin{array}{c} M_{k,j}^{\text{PPM}}=\frac{\;M_{k,j}^{\text{PFM}}+\left(p/4\right)}{N+p}. \end{array}$$

In order to prevent the matrix entries from appearing 0, according to human experience, the pseudocount [19] *p* was set 0.8, so that PPM was regarded as a part of the node features.

### 2.3. Model Architecture

Although traditional convolution techniques perform well for Euclidean data, they perform poorly for non-Euclidean data [20]. Therefore, graph convolution technology came into being. For a graph, the edges of each node are related to other nodes and this information can be used to capture interdependencies between instances, so the node can aggregate its own features and its neighbor features to generate a new representation of the node [21]. With the continuous development of graph learning, there are many variations, like GAT, GAE, and GGN [22]. All these network models can extract the feature; for using the GCN layer, each layer convolution operation is as shown in Equation (3):

(3) $$\begin{array}{c} H^{l+1}=f\left(H^{l},A\right)=\sigma \left({\overset{\wedge }{D}}^{-1/2}\hat{A}{\overset{\wedge }{D}}^{-1/2}H^{l}W^{l+1}\right). \end{array}$$

**Figure 3:**
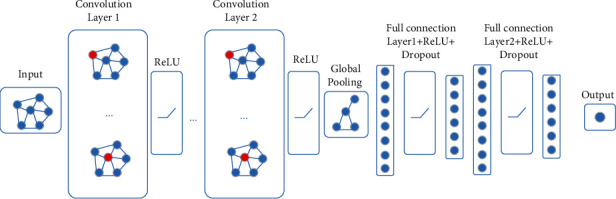
The structure of the GCN network, graphs of DNA-binding proteins through the GCN to get their representation.

Among them, *A* is the adjacency matrix of node features, assuming that the node number is *m*, then its adjacency matrix is (*m*, *m*), $\hat{D}$ is the degree of matrix (*m*, *m*), which represents the connection relationship between residues, $\hat{D}=D+I$, *I* is a unit matrix, considers itself features, *W*^*l*+1^ is the first *l* + 1 layer of weighting matrix, *H*^*l*^ is the output of the first layer of l, and *H*^0^ = *X*, *X* is the input of the feature matrix, Figure 3 shows the architecture of the model.

The protein graph contained much information about the interactions and positions of each residue pair, which was important for feature learning and predicting DNA-binding proteins. It was input into the GCN to extract the features. After convolution of multiple GCN layers, the representation of protein was effectively extracted. Then, the overall features of protein for prediction were obtained. The prediction includes two full connection layers. The results were presented as probabilities.

Using GCN to map proteins to the representation of rich features has also become a method of protein feature extraction. In addition, there were many factors affecting the experimental results, such as dropout, epoch, and batch. The setting of some hyperparameters were compared and determined through experiments.

## 3. Results and Discussion

The experiment was built on PyTorch [23], an open source deep learning framework. The GCN model was based on its PyG implementation [24], PDB14189 was used for testing to find the optimal super parameters, and PDB2272 was used to test model performance.

### 3.1. The Evaluation Index

Accuracy (ACC), Matthews correlation coefficient (MCC), sensitivity (SN), and specificity (SP) were used as the evaluation indexes of the model [25], these indexes were widely used in the studies of biological sequences, as shown in

(4) $$\begin{array}{c} \left\{\begin{array}{c} \text{SN}=\frac{\text{TP}}{\text{TP}+\text{FN}},\\ \text{SP}=\frac{\text{TN}}{\text{TN}+\text{FP}},\\ \text{ACC}=\frac{\text{TP}+\text{TN}}{\text{TP}+\text{FP}+\text{TN}+\text{FN}},\\ \text{MCC}=\frac{\text{TP}\times \text{TN}-\text{FP}\times \text{FN}}{\sqrt{\left(\text{TP}+\text{FN}\right)\times \left(\text{TN}+\text{FP}\right)\times \left(\text{TP}+\text{FP}\right)\times \left(\text{TN}+\text{FN}\right)}}. \end{array}\;\right. \end{array}$$

Among them, TP is the number of the correctly predicted positive samples, TN is the number of the correctly predicted negative samples, FP is the number of the wrongly predicted positive samples, and FN is the number of the wrongly predicted negative samples. SN represents the percentage of correctly predicted positive samples, SP represents the percentage of correctly predicted negative samples, ACC represents the percentage of correctly predicted samples in total samples, and MCC represents the prediction quality of the binary classification model, with a range of [−1, 1]. The larger the MCC is, the better the prediction quality of the model is.

### 3.2. The Setting of Hyperparameters

Training an optimal model requires constantly adjusting the hyperparameters of the model, which can be modified based on human experience. Some of the hyperparameters were shown in Table 3. In this model, according to human experience, the GCN layer was set to three, dimensions of input and output for each layer were shown in Table 4. Some other parameters were compared in the following experiences.

### 3.3. Model Performance when Selecting Different Dropouts

**Figure 4:**
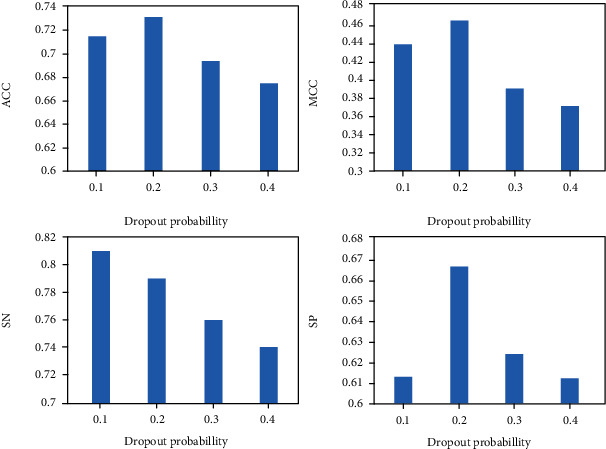
Comparison of prediction performance of different dropout probabilities.

After protein feature extraction, in order to better improve the accuracy of classification, two full connection layers were added to the ends to improve the learning ability of the model. In the fully connected layer, in order to avoid overfitting of the model, dropout was introduced to shut down some neurons with a probability value. Different probability values will affect the performance of prediction. To evaluate the impact of different dropout values, Figure 4 shows the performance of the model according to different dropout values. When the dropout is 0.2, the model has the highest performance compared to other parameters.

### 3.4. Whether PSSM Is Included in Feature Selection

**Figure 5:**
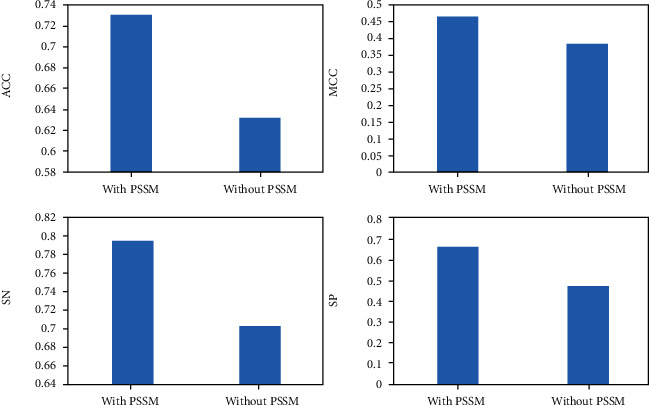
Comparison of performance results with or without PSSM.

The selection of protein feature greatly affects the accuracy of prediction. Since the dimension of PSSM matrix constructed by features was very small, the experiment was carried out with PSSM or without PSSM. Figure 4 shows the results of various indicators under the condition. PSSM depends on the sequence correlation results, which contains much evolutionary information about the sequence, and ultimately determines the protein features. As can be seen from Figure 5, PSSM can effectively represent the features of proteins and effectively improve the prediction performance.

### 3.5. Analysis of Experimental Results

In the independent test dataset, PDB14189 was used as the training dataset to train the model, and PDB2272 was used as the test dataset. According to the optimal experimental parameters, the final DNA-binding protein classification model was constructed: the number of GCN layers were three, dropout was 0.2, PSSM was selected as the feature, the input and output dimensions of each layer were (54, 54), (54,108), and (108,216). Other methods were compared with the method, and the method reached ACC (78.49%), SN (92.59%), SP (64.15%), and MCC (59.27%). Under certain conditions, the method has certain advantages compared with the existing methods, as shown in Table 5.

## 4. Conclusions

DNA-binding proteins are enzymes, which can bind with DNA to produce complex proteins and play important roles in the functions of a variety of biological molecules. In order to improve the accuracy of prediction of DNA-binding protein, a DNA-binding protein prediction model based on GCN and contact map was proposed. In this model, the dataset was preprocessed by sequence alignment; then, the structural information is extracted by Pconsc4 model; PSSM and some biological characteristics are used as features. Finally, the GCN model was constructed to train and predict DNA-binding protein data. The protein graph contained information about the interactions and positions of each residue pair, which was important for feature learning and predicting binding proteins. The protein graph was input into the GCN to extract the features, and the prediction included two full connection layers. Using GCN to map proteins to the representation of rich features has also become a method of protein feature extraction. Through training and parameter tuning, the performance of GCN model was better than some existing methods. It also provides some thoughts for other fields of biological information.

In the future, we plan to carry out a research on feature extraction and network model to improve the accuracy of DNA-binding proteins and related prediction. Different biological features can be combined, and methods such as attention mechanism can be considered to improve the model, in order to achieve the goal of improving the prediction effect and other indicators.

## Figures and Tables

**Table 1:** Introduction to the dataset.

Number\dataset	PDB14189	PDB2272
Positive	7129	1153
Negative	7060	1119
Total	14189	2272

**Table 2:** Node features.

Label	Feature	Size
1	One-hot encoding of the residue symbol	21
2	Position-specific scoring matrix (PSSM)	21
3	Whether the residue is aliphatic	1
4	Whether the residue is aromatic	1
5	Whether the residue is polar neutral	1
6	Whether the residue is acidic charged	1
7	Whether the residue is basic charged	1
8	Residue weight	1
9	The negative of the logarithm of the dissociation constant for the –COOH group	1
10	The negative of the logarithm of the dissociation constant for the –NH3 group	1
11	The negative of the logarithm of the dissociation constant for any other group in the molecule	1
12	The pH at the isoelectric point	1
13	Hydrophobicity of residue (pH = 2)	1
14	Hydrophobicity of residue (pH = 7)	1
	Total	54

**Table 3:** The hyperparameter settings using human experience.

Hyperparameter	Setting
Epoch	1000
Batch size	128
Learning rate	0.001
Optimizer	Adam
The number of convolution layers	3
Fully connected layers after GCN	2

**Table 4:** Combinations of GCN models on PDB14189.

Model	Number of layers	Layer1 (in, out)	Layer2 (in, out)	Layer3 (in, out)
GCN	1	(54,54)	—	—
GCN	2	(54,54)	(54,108)	—
GCN	3	(54,54)	(54,108)	(108,216)

**Table 5:** Comparison between the proposed method and existing methods on PDB2272.

Methods	ACC (%)	MCC (%)	SN (%)	SP (%)
Qu et al. [26]	48.33	3.34	48.31	48.35
Local-DPP [27]	50.57	4.56	8.76	93.66
Pse-DNA-Pro [28]	61.88	24.30	75.28	48.08
DPP-Pse-AAC [29]	58.10	16.25	56.63	59.61
Ms-DBP [30]	66.99	33.97	70.69	63.18
GCN-method	78.49	59.27	92.59	64.15

## Data Availability

The datasets can be found in the references.
